# Supplementary material for: Validation of the Measuring Protocol for the Infraspinatus Muscle with M-Mode Ultrasound in Asymptomatic Subjects. Intra- and Inter-examiner Reliability Study
Source: Diagnostics (Basel). 2023 Feb 4;13(4):582. doi: 10.3390/diagnostics13040582 (PMC9955823; doi:10.3390/diagnostics13040582)
Supplement: Supplementary file 1 [file diagnostics-13-00582-s001.zip › diagnostics-2201875-supplementary.pdf]

Supplementary material. Table S1: Baseline outcomes vs. ultrasound measurement correlations.

Comparison	Correlation coefficient (95%CI)	[a]p value
Activation velocity vs. Age	0.431 (0.199, 0.617)	0.001
Activation velocity vs. BMI	0.305 (0.056, 0.519)	0.018
Activation velocity vs. Dominant upper limb	0.405 (-0.289, 0.82)	0.192
Activation velocity vs. Gender	-0.235 (-0.532, 0.113)	0.167
Activation velocity vs. Height	0.119 (-0.139, 0.362)	0.366
Activation velocity vs. Observed upper limb	-0.023 (-0.331, 0.29)	0.891
Activation velocity vs. Sport activity	-0.109 (-0.42, 0.226)	0.525
Activation velocity vs. Weight	0.327 (0.079, 0.536)	0.011
Activation velocity vs. Work activity	0.355 (0.013, 0.622)	0.026
MVIC vs. Age	0.328 (0.081, 0.537)	0.01
MVIC vs. BMI	0.303 (0.053, 0.517)	0.019
MVIC vs. Dominant upper limb	0.579 (0.02, 0.862)	0.008
MVIC vs. Gender	-0.458 (-0.686, -0.147)	0.001
MVIC vs. Height	0.275 (0.023, 0.495)	0.033
MVIC vs. Observed upper limb	-0.131 (-0.419, 0.181)	0.405
MVIC vs. Sport activity	0.278 (-0.164, 0.627)	0.19
MVIC vs. Weight	0.4 (0.162, 0.593)	0.002
MVIC vs. Work activity	0.266 (-0.052, 0.535)	0.083
Relaxation velocity vs. Age	0.348 (0.103, 0.553)	0.006
Relaxation velocity vs. BMI	0.284 (0.033, 0.502)	0.028
Relaxation velocity vs. Dominant upper limb	0.443 (-0.255, 0.837)	0.142
Relaxation velocity vs. Gender	-0.054 (-0.362, 0.265)	0.746
Relaxation velocity vs. Height	0.095 (-0.163, 0.341)	0.47
Relaxation velocity vs. Observed upper limb	-0.003 (-0.312, 0.307)	0.986
Relaxation velocity vs. Sport activity	-0.2 (-0.494, 0.134)	0.226
Relaxation velocity vs. Weight	0.295 (0.044, 0.511)	0.022
Relaxation velocity vs. Work activity	0.323 (-0.017, 0.596)	0.045
Thickness at rest vs. Age	0.265 (0.012, 0.486)	0.041

Supplementary material. Table S1: Baseline outcomes vs. ultrasound measurement correlations.

Comparison	Correlation coefficient (95%CI)	[a]p value
Thickness at rest vs. BMI	0.41 (0.174, 0.601)	0.001
Thickness at rest vs. Dominant upper limb	0.436 (-0.078, 0.767)	0.053
Thickness at rest vs. Gender	-0.495 (-0.701, -0.214)	<0.001
Thickness at rest vs. Height	0.304 (0.055, 0.518)	0.018
Thickness at rest vs. Observed upper limb	-0.171 (-0.454, 0.143)	0.275
Thickness at rest vs. Sport activity	0.193 (-0.184, 0.521)	0.303
Thickness at rest vs. Weight	0.488 (0.268, 0.66)	<0.001
Thickness at rest vs. Work activity	0.293 (-0.019, 0.553)	0.05
Thickness in contraction vs. Age	0.308 (0.059, 0.521)	0.017
Thickness in contraction vs. BMI	0.386 (0.147, 0.583)	0.002
Thickness in contraction vs. Dominant upper limb	0.448 (-0.064, 0.774)	0.044
Thickness in contraction vs. Gender	-0.491 (-0.697, -0.211)	<0.001
Thickness in contraction vs. Height	0.282 (0.03, 0.5)	0.029
Thickness in contraction vs. Observed upper limb	-0.102 (-0.397, 0.213)	0.526
Thickness in contraction vs. Sport activity	0.176 (-0.198, 0.505)	0.348
Thickness in contraction vs. Weight	0.46 (0.233, 0.639)	<0.001
Thickness in contraction vs. Work activity	0.313 (0.003, 0.569)	0.034
Time vs. Age	0.351 (0.107, 0.556)	0.006
Time vs. BMI	0.129 (-0.129, 0.371)	0.327
Time vs. Dominant upper limb	0.295 (-0.166, 0.65)	0.179
Time vs. Gender	-0.041 (-0.346, 0.272)	0.802
Time vs. Height	0.044 (-0.213, 0.294)	0.74
Time vs. Observed upper limb	-0.221 (-0.493, 0.089)	0.147
Time vs. Sport activity	0.041 (-0.329, 0.4)	0.835
Time vs. Weight	0.127 (-0.131, 0.369)	0.335
Time vs. Work activity	0.275 (-0.035, 0.537)	0.066

95%CI: 95% confidence interval.

[a]significant if p<0.05.
